# Genome-wide identification of histone methylation (H3K9_me2_) and acetylation (H4K12_ac_) marks in two ecotypes of switchgrass (*Panicum virgatum* L.)

**DOI:** 10.1186/s12864-019-6038-x

**Published:** 2019-08-22

**Authors:** Vasudevan Ayyappan, Venkateswara R. Sripathi, Venu ( Kal) Kalavacharla, Malay C. Saha, Jyothi Thimmapuram, Ketaki P. Bhide, Elizabeth Fiedler

**Affiliations:** 1 Molecular Genetics and Epigenomics Laboratory, College of Agriculture and Related Sciences, Delaware State University, Dover, DE USA; 2 Molecular Biology and Bioinformatics Laboratory, College of Agricultural, Life and Natural Sciences, Alabama A&M University, Normal, AL USA; 3 Center for Integrated Biological and Environmental Research, Delaware State University, Dover, DE USA; 4 Noble Research Institute, Ardmore, OK USA; 5 Bioinformatics Core, Purdue University, West Lafayette, IN USA

**Keywords:** Switchgrass, Epigenome, ChIP-Seq, Histone modifications, Differential binding, Phenylpropanoid pathway, And monolignols

## Abstract

**Background:**

Histone modifications play a significant role in the regulation of transcription and various biological processes, such as development and regeneration. Though a few genomic (including DNA methylation patterns) and transcriptomic studies are currently available in switchgrass, the genome-wide distribution of histone modifications has not yet been studied to help elucidate gene regulation and its application to switchgrass improvement.

**Results:**

This study provides a comprehensive epigenomic analyses of two contrasting switchgrass ecotypes, lowland (AP13) and upland (VS16), by employing chromatin immunoprecipitation sequencing (ChIP-Seq) with two histone marks (suppressive- H3K9_me2_ and active- H4K12_ac_). In this study, most of the histone binding was in non-genic regions, and the highest enrichment was seen between 0 and 2 kb regions from the transcriptional start site (TSS). Considering the economic importance and potential of switchgrass as a bioenergy crop, we focused on genes, transcription factors (TFs), and pathways that were associated with C4-photosynthesis, biomass, biofuel production, biotic stresses, and abiotic stresses. Using quantitative real-time PCR (qPCR) the relative expression of five genes selected from the phenylpropanoid-monolignol pathway showed preferential binding of acetylation marks in AP13 rather than in VS16.

**Conclusions:**

The genome-wide histone modifications reported here can be utilized in understanding the regulation of genes important in the phenylpropanoid–monolignol biosynthesis pathway, which in turn, may help understand the recalcitrance associated with conversion of biomass to biofuel, a major roadblock in utilizing lignocellulosic feedstocks.

**Electronic supplementary material:**

The online version of this article (10.1186/s12864-019-6038-x) contains supplementary material, which is available to authorized users.

## Background

In eukaryotes, gene expression is a complex, coordinated process orchestrated by transcription factors (TFs) and chromatin proteins. As a result, chromosomal DNA is either compacted or relaxed to repress or activate the transcription process, respectively. At the whole-genome level, genetic and epigenetic factors together regulate gene expression. The accessibility of DNA sequences and chromatin sites associated with nucleosome packaging is known to determine transcription efficiency. Epigenetic factors such as DNA methylation, histone modifications, and chromatin remodelors play a significant role in the regulation of gene expression and various biological processes, such as development [1], regeneration [2], and oncogenesis [3]. In plants, with recent advances, genome-wide epigenomic analysis methods are increasingly supporting the role of chromatin modifications in gene expression [4].

Transcription factors are important in gene regulation; they bind to cis-regulatory sequences in the promoter region of their target genes to fine-tune gene expression [5]. In plants, transcription factors and histone modifications demonstrably play a role in development, vernalization, and response to biotic and abiotic stressors. [6–10]. The application of high-throughput technologies such as chromatin immunoprecipitation followed by genome-wide sequencing (ChIP-Seq) provides an opportunity to identify binding patterns for a protein(s) of interest in the entire genome. Genome-wide association and correlation of modifications, such as histone methylation and acetylation with biotic and abiotic stress responses in plants have been reported [8, 11–13].

Switchgrass (*Panicum virgatum* L.) is a native perennial and warm-season grass, widely used for biofuel, livestock feed, erosion control, and wildlife habitat. Carbon sequestration by switchgrass, a C4 plant with high cellulosic conversion rates and drought tolerance, facilitates its use as a biofuel directly or by ethanolic conversion, making it a valuable, natural renewable energy resource [14]. To date, studies in switchgrass have pertained to molecular breeding [15, 16], genomic [17, 18] and transcriptomic [19, 20] analyses, and response to biotic and abiotic [21, 22] stresses. Using Bisulfite sequencing (BS-Seq) and Methylated DNA immunoprecipitation sequencing (MeDIP-Seq), similar DNA methylation patterns between AP13 and VS6 have been revealed, and the methylation levels were higher in Transposable elements (TEs) when compared to genic regions [23]. The recent bisulfite sequencing study suggested small interfering RNAs (siRNAs) positively regulate DNA methylation and thereby interferes with gene and long non-coding RNAs (lncRNAs) expression in switchgrass [24]. However, the genome-wide distribution of histone modifications in switchgrass has not been reported. The histone marks (a repressive mark, H3K9_me2_, and an activation mark, H4K12_ac_) utilized in this study have been studied and reported previously in several plant species [7, 8, 25, 26]. Methylation of histone H3K9 is preferentially localized to heterochromatin in Arabidopsis [25], and its profiling has been reported in other plants, for instance, rice [7], maize [26], and recently in the common bean [8]. On the other hand, H4K12_ac_ is primarily localized in active coding regions of the genome and therefore creates binding sites for regulatory factors to promote transcription [8].

Switchgrass has two main ecotypes, lowland and upland, with distinct morphological and genetic characteristics and specific geographical niches [27]. Two important switchgrass genotypes (AP13 and VS16) were selected to represent each of these two contrasting ecotypes. Among these, AP13, a lowland ecotype, was used for the developing the switchgrass reference genome [28]. A female parent AP13 and male parent VS16 (an upland ecotype) were utilized for the construction of a genetic linkage map [18] and the identification of quantitative trait loci (QTL) associated with biomass yield and traits related to recalcitrance [22, 29]. Due to their adaptability and ability to withstand selection pressure from diverse environmental stressors, the inherent genetic potential of these genotypes at the level of gene expression has been recently explored [30]. However, little is known about the epigenetic regulation of gene expression associated with these genotypes. The H3K9_me2_ and H4K12_ac_ marks selected in this study regulate the closed and open chromatin states, respectively, during transcription. In this study, we report the first genome-wide profiling of histone methylation H3K9_me2_ and histone acetylation H4K12_ac_ marks by using ChIP-Seq in upland (VS16) and lowland (AP13) switchgrass ecotypes.

## Methods

### Plant materials and sample collection

The two contrasting, heterozygous, tetraploid genotypes (2n = 4x = 36) utilized in this study (AP13 and VS16) were derived from two ecotypes of switchgrass: the lowland cultivar, Alamo and the upland cultivar, Summer, respectively. The leaf materials were obtained from our collaborator and co-author on this manuscript Malay Saha at the Noble Research Institute (Ardmore, OK). Both AP13 and VS16 actively grow in summer, and hence the conditions (29/22 °C day/night temperatures and a 16 h photoperiod) considered in the greenhouses of the Noble Research Institute were uniform. Fully expanded leaves were randomly collected and pooled from five plants of one-month-old clonal ramets, cryopreserved in liquid nitrogen, and stored at − 80 °C. This experiment included two genotypes (AP13 and VS16), two marks (H3K9_me2_ and H4K12_ac_), two conditions (input control and actual sample), and three replicates resulting in (2 X 2 X 2 X 3) a total of 24 samples. Of significance is that this current study on global ChIP-seq analyses utilized the same leaf samples that were collected, stored (− 80 °C), and used for RNA-seq analyses in our previous studies [30]. Since our goal is to understand the genome-wide histone modifications in regulating global gene expression rather than a specific focus on pathways related to lignin formation, we have used leaves instead of stems.

### Isolation and immunoprecipitation of chromatin

All the lyophilized leaf samples were ground to a fine powder in liquid nitrogen, collected in frozen 50 mL falcon tubes (Corning Inc., Corning, NY). Next, 25 ml (mL) of cold nuclear isolation buffer containing 1% formaldehyde, and 20 μl (μl) of proteinase inhibitor (Thermo Scientific, IL, USA, Product #87786) were added. The samples were incubated for 5 min at room temperature. Glycine (2 M) was used to terminate the crosslinking reaction. The lysate was filtered before nuclei pelletization at 3000 g for 10 min at 4 °C. The pellet was resuspended in cold nuclear isolation buffer (no formaldehyde included). An equal volume of 15% Percoll solution was added to the lysate, and the nuclei were pelleted again at 3000 g for 5 min at 4 °C. The pellet was then mixed in nuclear lysis buffer. This lysate was then sonicated using a Soniprep 150 (MSE UK Ltd., London, UK) for 6 pulses with 15 s between each pulse. The sheared DNA fragments collected after sonication ranged between 100 and 350 bp when visualized by agarose gel electrophoresis. ChIP dilution buffer was then added to the nuclear lysate to attain 10X dilution, and chromatin samples were then divided equally into two tubes. One of the samples was incubated with rabbit polyclonal antibody in a 20:1 mixture that specifically adhered to the N-terminus of histone subunit H4 acetylated at lysine 12 (H4K12_ac_) [31]. Also, the chromatin sample was incubated with a mouse antibody in a 20:1 mixture, which adhered precisely to the epitope region of histone H3 di-methylated at lysine 9 (H3K9_me2_) [32]. Twenty microliters of Pierce Protein A/G magnetic beads (ThermoFisher Scientific, Grand Island, NY) were added to the nuclear lysate. The samples were then incubated overnight at 4 °C. The chromatin samples that had no antibodies served as positive controls. Later, the centrifuged and pelleted magnetic beads were washed thrice with 100 μl ChIP dilution buffer. The magnetic beads bound to chromatin and antibody complex were then eluted with freshly prepared elution buffer. To terminate the crosslinking, 20 μl of 5 M NaCl was added and subsequently incubated at 65 °C overnight. After this, 5 μl of 2 M Proteinase-K (Thermo Scientific) was added and incubated for 2 h (h) at 45 °C. DNA was then collected by phenol-chloroform extraction followed by ethanol precipitation. The ChIP-DNA was resuspended in 20 μl of TE buffer. After verification by ChIP-PCR, each of these purified DNA samples were then used to generate a ChIP-Seq library using Illumina HiSeqTM 2500 (Illumina Inc., San Diego, CA). Libraries were sequenced at the Delaware Biotechnology Institute (DBI, Newark, DE).

### Library construction and sequencing

The quality, purity, and size of the immunoprecipitated DNA samples were determined by an AATI Fragment Analyzer (Advanced Analytical, Ames, IA). The ChIP-Seq libraries (101 bp paired-end) were prepared by using Illumina TruSeq ChIP Sample Preparation kit (IP-202-1012; Illumina Inc., San Diego, CA). The NGS data generated in this study was submitted to the SRA section of NCBI with the project number PRJNA373877 (https://www.ncbi.nlm.nih.gov/bioproject/?term=PRJNA373877).

### ChIP-Seq data analysis workflow

Sequence quality was assessed using FastQC (v0.11.2) [33] and quality trimming was carried out using the FASTX toolkit (v0.0.13) (http://hannonlab.cshl.edu/fastx_toolkit) to filter bases with less than the Phred33 score of 30 and to remove sequences with less than 50 bases. The quality trimmed reads were mapped to the AP13 v1.1 reference genome, *Panicum virgatum*, (https://phytozome.jgi.doe.gov/pz/portal.html#!info?alias=Org_Pvirgatum), using bowtie2 (v2.2.6) [34] with default parameters, except the number of mismatches was set to 1. Peaks marked by H3K9_me2_ and H4K12_ac_ were identified using Spatial Clustering for Identification of ChIP-Enriched Regions (SICER; v1.1) [35] for both genotypes, using the following parameters: redundancy threshold of 10; window size of 200; gap size of 600; effective genome size as a fraction of reference genome of 0.7; and false discovery rate (FDR) threshold controlling significance at 0.01, for each replicate/sample with respect to their input control. The set of peaks present in at least two replicates was used for further analysis. A gene was considered methylated (H3K9me2-marked) or acetylated (H4K12ac-marked) only if it was overlapping (based on known annotated genes) with peak coordinates by at least one base. To study the pattern of histone binding in different regions of the genome, we also calculated distribution of peaks identified in different genomic regions (5′ UTR, exon, intron, 3′ UTR, upstream, downstream, and non-genic regions using PAVIS (v1.8) [36] based on the annotation of known genes. For each peak, the distance from the peak to the TSS was determined and plotted using annotations from Phytozome for the AP13 v1.1reference genome. The percentage of genes for each modification was calculated as the total number of genes with peaks divided by the number of genes for each modification and multiplied by 100. As routinely used in expression profiling of genes associated with histone peaks in other biological systems, once we obtained the nearest annotated genes, we performed Gene Ontology (GO) analysis to assign gene functions such as biological processes, molecular functions, and cellular components, before conducting Kyoto Encyclopedia of Genes and Genomes (KEGG) pathway analysis. Among thousands of genes that were differentially marked or enriched using ChIP-Seq analysis, five genes (1–5) were selected for functional validation based on: i) their role in phenylpropanoid-monolignol pathway; ii) fold change (Log2FC) > 2; and iii) FDR < 0.01.

### ChIP-quantitative real time (RT)-PCR (qPCR)

To quantitatively measure the amplification of ChIP-DNA, quantitative real-time (RT)-PCR (qPCR) validation was performed with an ABI 7500 real-time PCR (Applied Biosystems, Foster City, CA). The five genes selected here belong to the phenylpropanoid pathway that represents the active (H4K12_ac_) and inactive (H3K9_me2_) modifications from ChIP-Seq analysis. The primers were designed using the TaqMan qPCR primer design tool (GenScript USA Inc., Piscataway, NJ), and the primer pairs used to amplify the ChIP-enriched regions are presented in Additional file 1: Table S1. qPCR was carried out in a 25 μL reaction that contained 10 ng of immunoprecipitated DNA, 10 μM each of forward and reverse primers, and 12.5 μl of SYBR Green PCR Master Mix (Germantown, MD). The qPCR reaction settings were: 95 °C for 10 min, followed by 40 cycles of 95 °C for 15 s and 65 °C for 1 min.

We used three replicates to test the repeatability of qPCR validations. The universal cons7 primer was used as a constitutive control to determine normalized expression for all samples. Primer efficiency was determined by using 2-ΔΔCT method [37], where ΔΔCT (CT of genes - CT of cons7) = tissue to be observed - leaf tissue (CT of genex - CT of cons7). Minitab 17 [38] was used to analyze the normalized CT values (ΔΔCT), and the expression results were presented as mean ± SE. To test the multiple comparisons between the sample means, one-way ANOVA was conducted on normalized CT values collected from qPCR analysis.

## Results

Utilizing AP13 v1.1 as a reference [28], 138 million and 114 million clean 101 bp paired-end reads were obtained for H3K9_me2_ and H4K12_ac_ modifications for AP13, respectively. We also obtained 120 million and 108 million clean 101 bp paired-end reads marked respectively with H3K9_me2_ and H4K12_ac_ for VS16. Average mapping rates of 82.32% (H3K9_me2_) and 81.20% (H4K12_ac_) in AP13 and 73.42% (H3K9_me2_) and 68.43% (H4K12_ac_) in VS16 (Table 1), respectively, were observed.

**Table 1:** Statistics of ChIP sequencing of switchgrass genotypes AP13 and VS16 and their alignments to the reference genome AP13 v1.1 (*P. virgatum*)

Sample	Total Reads	Quality Control Reads	% Reads Passing QC	Total Reads Mapped	% Reads Mapped
AP13H3	138,072,842	132,672,004	96.08	113,596,549	82.32
AP13H3 Input	37,218,084	36,739,685	98.71	34,351,304	92.29
VS16H3	119,727,844	115,043,859	96.05	87,947,726	73.42
VS16H3 Input	44,430,086	43,948,490	98.91	35,837,847	80.66
AP13H4	113,969,918	108,106,092	94.9	92,471,201	81.20
AP13H4 Input	42,305,540	41,843,913	98.9	39,138,138	92.51
VS16H4	107,606,766	103,864,501	96.5	73,455,893	68.43
VS16H4 Input	48,779,324	48,779,324	99.02	39,773,125	81.53

### Distribution of histone modifications

**Fig. 1:**
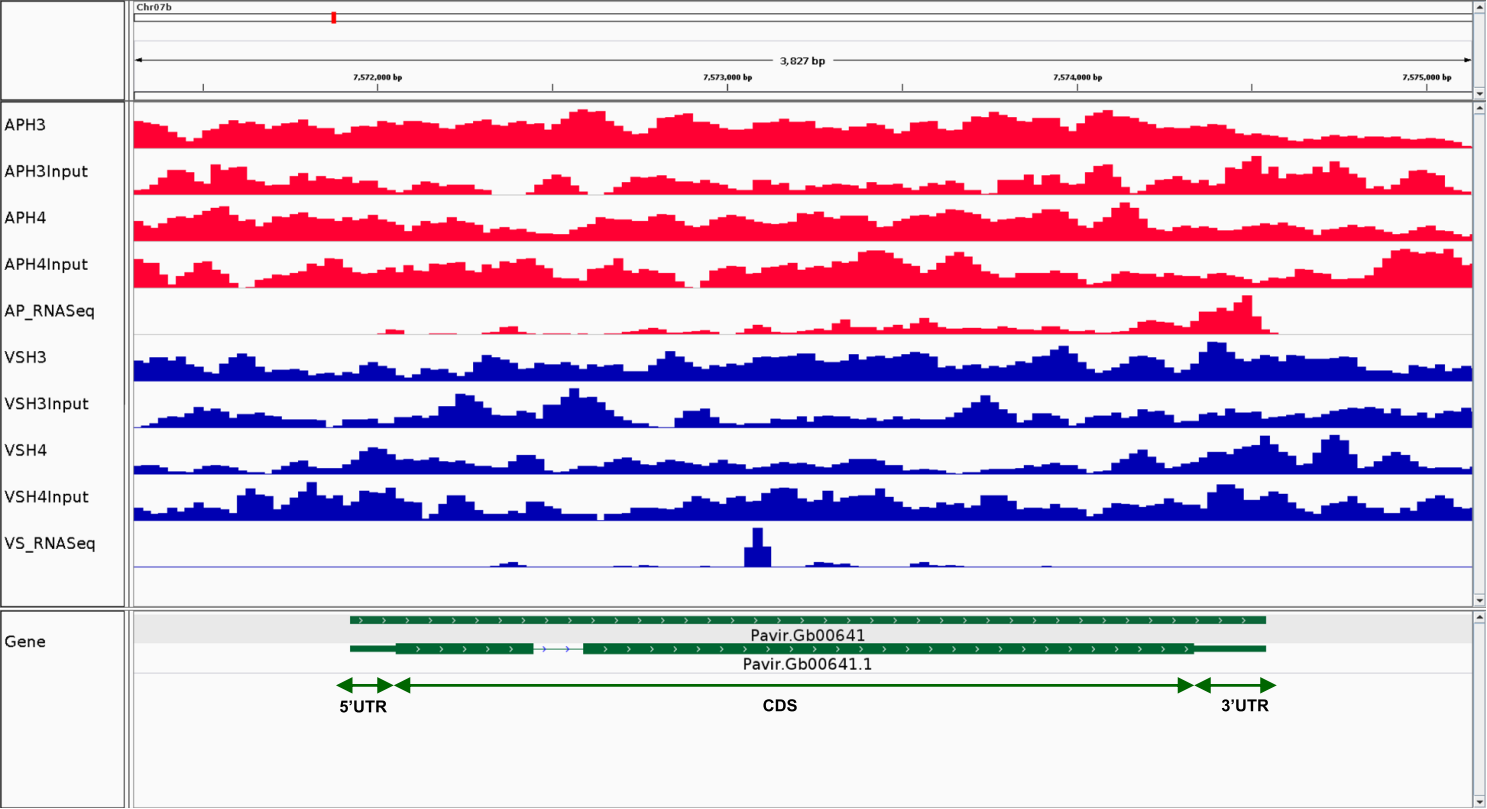
Comparative visualization of a representative region of Phenylalanine Ammonia Lyase (PAL) gene on chromosome 7b in switchgrass genotypes AP13 (red) and VS16 (blue) with INPUT (positive control) as background for (A) H3K9_me2_ and (B) H4K12_ac_ modification using Integrative Genomics Viewer (IGV). R1-R3 represents the three biological replicates

**Fig. 2:**
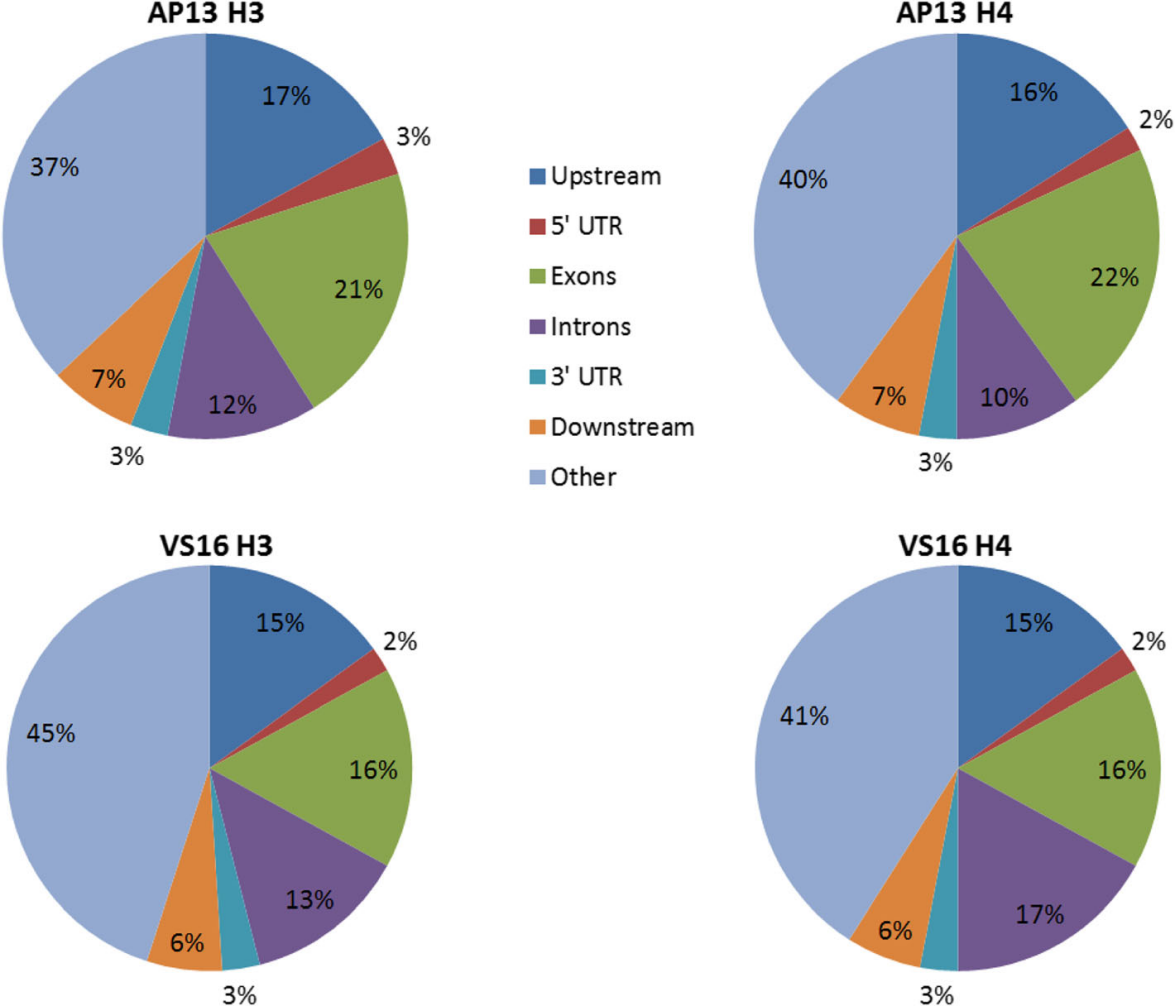
Distribution of H3K9_me2_ and H4K12_ac_ peaks in genic and non-genic regions of switchgrass genotypes AP13 and VS16

Overall distributions of H3K9_me2_ and H4K12_ac_ peaks were obtained using reads that mapped to all chromosomes and contigs greater than 5 kb, and contigs between 2 kb and 5 kb that have predicted genes while calling peaks (Additional file 2: Figure S1). A representative graph that shows the distribution of histone marks on phenylalanine ammonia lyase (PAL) gene in chromosome 7b was visualized in AP13 and VS16 for H3K9_me2_ and H4K12_ac_ modifications, using input (AP13 v1.1) as a positive control through the Integrative Genomics Viewer (IGV) [39] (Fig. 1). For H3K9_me2_ and H4K12_ac_ in both AP13 and VS16, numerous peaks were identified in non-genic regions (over 40%), and less than 40% of peaks were in genic regions (Fig. 2). The range of non-genic region for AP13 for H3K9_me2_ (399 bp - 27,799 bp) and H4K12_ac_ (399 bp – 32,399 bp) modifications and VS16 for H3K9_me2_ (199–34,199) and H4K12_ac_ (199 bp – 29,399 bp) modifications has been observed in this study.

### Genes related to peaks

By analyzing genes within 10 kb from the peaks, 24,163 and 21,918 genes were identified in AP13 and VS16, respectively. Of these, 5852 (AP13) and 3918 (VS16) gene-associated peaks were marked with H3K9_me2_, while 18,311 (AP13) and 18,000 (VS16) gene-associated peaks were marked with H4K12_ac_ (Table 2). In AP13 and VS16, the percentage of methylated peaks located on genic regions was 24.22 and 17.88%, respectively. Also, the percentage of acetylated peaks located on genic regions in AP13 and VS16 was 75.78 and 82.12%, respectively. Furthermore, 71.86 and 63.31% of these genes carried both of the modifications (H3K9_me2_ and H4K12_ac_) in AP13 and VS16, respectively (Table 2).

**Table 2:** Genes of AP13 and VS16 switchgrass genotypes associated with H3K9_me2_ and H4K12_ac_ regions^1^

Genotypes	Number of Genes associated with Peaks^1^	Genes with H3K9_me2_ (%)	Genes with H4K12_ac_ (%)	Genes with Both H3K9_me2_ and H4K12_ac_ (%)
AP13	24,163	5852 (24.22)	18,311 (75.78)	17,364 (71.86)
VS16	21,918	3918 (17.88)	18,000 (82.12)	13,878 (63.31)

^1^H3K9 di-methylation and H4K12 acetylation regions include 10 kb upstream and downstream from the peak start position

### Distances from peaks to TSSs

**Fig. 3:**
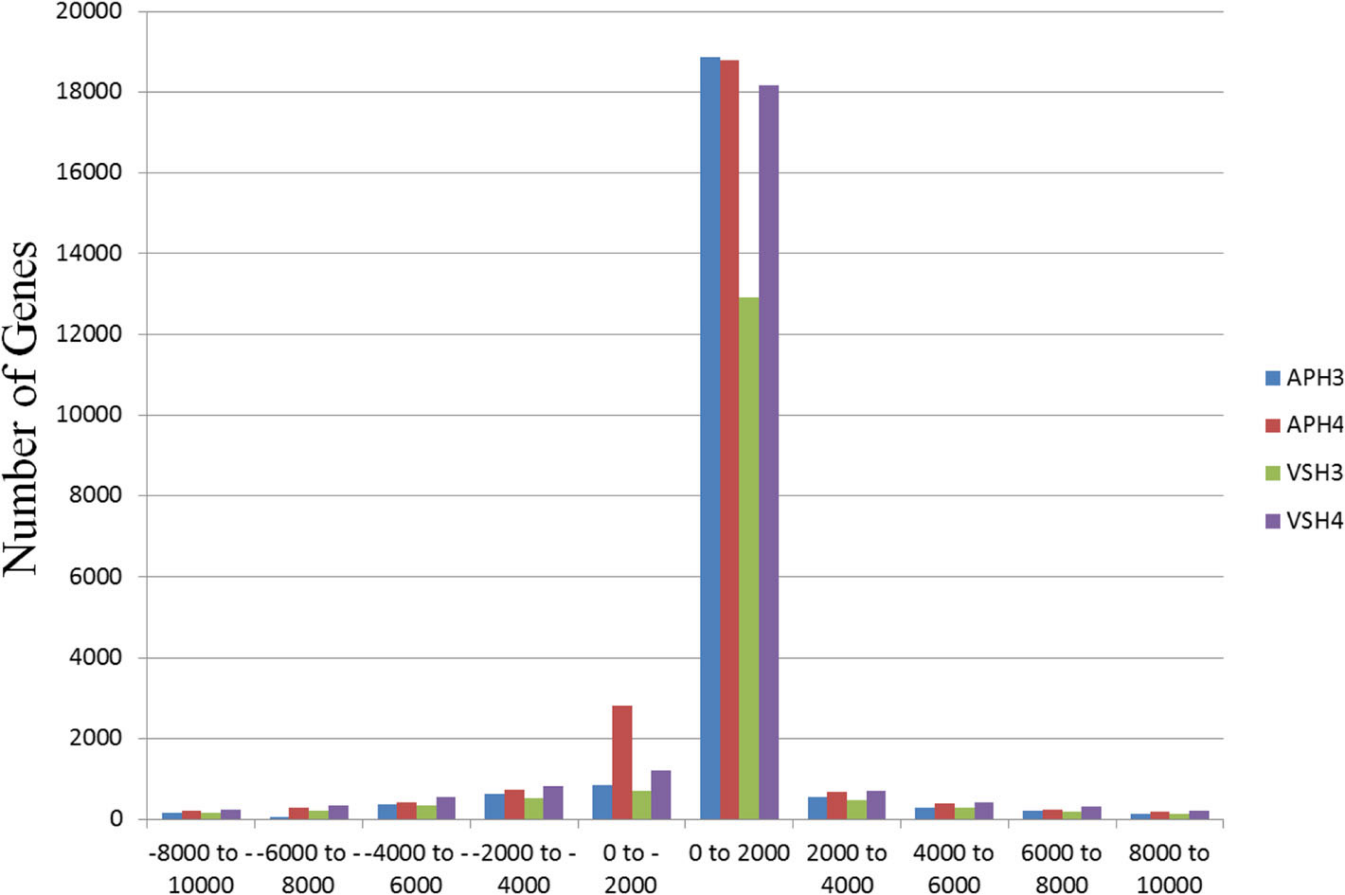
Distance of peaks to transcriptional start sites (TSSs) for H3K9_me2_ and H4K12_ac_ modifications in switchgrass genotypes AP13 and VS16

Based on nearest gene distribution using BEDTools (v2.21.0) [40], over 18,000 methylated and acetylated peaks spanned between 0 to + 2000 bases from TSSs in AP13. In addition, over 2000 acetylated peaks spanned between − 1 to − 2000 bases from TSSs in AP13. Over 12,000 methylated and 18,000 acetylated peaks spanned between 0 to + 2000 bases from TSSs in VS16. (Fig. 3). Most enrichment was seen in the region 2 kb upstream or downstream of a TSS. The enrichment levels from both upstream and downstream of 2 Kb from TSS was seen to gradually decrease in both modifications for both genotypes (Additional file 3: Figure S2A-D). To understand the relationship between histone modifications (H3K9_me2_ and H4K12_ac_) and TF binding, the peak distances to nearest genes were binned into 5 kb intervals (Additional file 4: Figure S3A-D). Of 30 bins with a nearest gene distance of 15 kb, 26 contain at least 100 peaks for H3K9_me2_ modification in AP13 and VS16 (Additional file 4: Figure S3A and B). All 30 bins with a nearest gene distance of 15 kb for the H4K12_ac_ modification contain at least 100 peaks for both genotypes (Additional file 4: Figure S3C and D).

### Epigenomic factors

The five major categories of epigenetic factors identified, along with the number of genes marked with H3K9_me2_ in both genotypes (AP13/VS16), were methyl groups (186/159), methyl CpG-Binding domains (73/69), acetyltransferase groups (36/39), oxidase/desaturase groups (14/16), and chromatin remodeling factors (21/41). Similarly, genes marked with H4K12_ac_ and their corresponding counts for methyl groups, methyl CpG-Binding domains, acetyltransferase groups, oxidase/desaturase groups, and chromatin remodeling factors were 196, 69, 39, 14 and 21 in AP13 and 135, 95, 68, 16 and 41 in VS16, respectively (Additional file 5: Figure S4). In this study, epigenomic factors, methyl CpG binding domains, and acetyltransferase groups were most dominant.

### Pathways and genes associated with peaks

Although several pathways and pathway-related genes were identified in the study, we only selected 16 pathways that had greater than or equal to10 genes associated with both H3K9_me2_ and H4K12_ac_ in each genotype (Table 3). Of these, secondary metabolite biosynthesis pathways were predominant, with over 100 genes methylated and acetylated in both AP13 and VS16 genotypes. Therefore, greater emphasis was placed on genes, pathways, and TFs associated with biomass/biofuel production as well as biotic and abiotic stresses. Functions for the nearest genes to these peaks were assigned using KEGG pathway analysis, and most of these genes were functionally associated with cellular and metabolic processes, responses to stimuli, other biological regulation, etc. (Additional file 1: Tables S2, S3, S4 and S5).

**Table 3:** KEGG pathway analysis for the genes with H3K9_me2_ and H4K12_ac_ associated-binding sites in switchgrass genotypes AP13 and VS16

Pathway Description	Number of genes			
	H3K9_me2_		H4K12_ac_	
	AP13	VS16	AP13	VS16
Metabolic pathways	308	279	284	382
Biosynthesis of secondary metabolites	177	150	165	209
Biosynthesis of antibiotics	83	74	66	103
Microbial metabolism in diverse environments	67	65	50	72
Carbon metabolism	45	44	29	52
Biosynthesis of amino acids	42	37	38	54
Plant hormone signal transduction	29	24	28	28
Starch and sucrose metabolism	21	15	16	17
RNA transport	36	32	34	44
Cell cycle	16	22	20	26
Plant-pathogen interaction	15	13	14	15
Carbon fixation	15	14	10	10
Phenylpropanoid biosysnthesis	14	11	12	11
Fatty acid metabolism	12	11	12	16
Terpenoid backbone biosynthesis	10	12	12	18
Photosynthesis	9	4	12	2

### C4 photosynthesis-related enzymes

An important intermediate in C4 carbon-fixation pathway, phosphoenol pyruvate (PEP) and two key pathway enzymes; i.e., phosphoenolpyruvate carboxylase (PEPC), that catalyzes PEP and carbonic anhydrase (CA), and two transporter genes viz., tonoplast dicarboxylate transporter (DIT) and bile acid sodium symporter/pyruvate transporter (BASS) were identified as marked in both AP13 and VS16 for both modifications, whereas thiophosphate isomerase was only identified in VS16 for both modifications (Table 4). The other genes, nicotinamide adenine dinucleotide phosphate (NADP)-dependent oxidoreductase, phosphoenolpyruvate carboxykinase (PEP-CK), and thiophosphate isomerase showed varied patterns in histone methylation and acetylation. For instance, in AP13, NADP-dependent oxidoreductase and PEP-CK were only methylated, while thiophosphate isomerase was neither methylated nor acetylated.

**Table 4:** List of C4-photosynthesis, photorespiration, and phenylpropanoid-monolignol pathway-related genes associated with H3K9_me2_ and H4K12_ac_ modifications in switchgrass genotypes AP13 and VS16

Genes Identified	Number of genes			
	H3K9_me2_		H4K12_ac_	
	AP13	VS16	AP13	VS16
C4-Related Photosynthetic Genes				
Tonoplast Dicarboxylate Transporter (DIT)	17	1	4	2
Phosphoenolpyruvate (PEP)	19	15	20	0
Phosphoenolpyruvate Carboxylase (PEPC)	9	9	10	10
Bile Acid Sodium Symporter/Pyruvate Transporter (BASS)	4	2	3	1
Carbonic Anhydrase (CA)	6	5	9	2
NADP-Dependent Oxidoreductase	11	3	0	0
Phosphoenolpyruvate Carboxykinase (PEP-CK)	1	0	0	1
Thiophosphate Isomerase	0	2	0	4
Photorespiration-Related Genes				
2-Oxoglutarate (2-OG)	100	57	91	96
Glutamate	59	46	49	61
3-Phosphoglycerate (3-PGA)	6	2	6	4
Ribulose 1,5-bisphosphate carboxylase/Oxygenase (RuBisCo)	4	3	7	5
Glutamine (GLN)	33	18	33	31
Dicarboxylate Transporter (DIT)	1	2	4	2
Catalase (CAT)	2	0	2	4
Glutamate Synthase (GS)	3	4	4	4
Phenylpropanoid-Monolignol Pathway-Related Genes				
Phenylalanine Ammonia Lyase (PAL)	11	15	27	4
4-Coumarate:CoA Ligase (4CL)	4	3	8	2
Hydroxycinnamoyl CoA:Shikimate Hydroxycinnamoyl Transferase (HCT)	32	17	32	25
Cinnamyl alcohol dehydrogenase (CAD)	33	26	42	31
Caffeoyl-CoA 3-O-Methyl Transferase (CCoAOMT)	6	1	5	1
Cinnamate 4-hydroxylase (C4H)	2	1	1	1

### Photorespiration pathway-related genes

Marked by H3K9_me2_ and H4K12_ac_ in both the genotypes were eight key photorespiratory enzymes: 2-oxoglutarate (2-OG), glutamate (GLU), 3-phosphoglycerate (3-PGA), ribulose 1,5-bisphosphate carboxylase/oxygenase (RuBisCo), glutamine (GLN), dicarboxylate transporter (DIT), catalase (CAT), and glutamate synthase (GS) (Table 4). Of these eight genes, 2-OG, GLU, and GLN showed binding affinity to H3K9_me2_ and H4K12_ac_ in both genotypes.

### Phenylpropanoid pathway-related genes

Utilizing shikimate pathway intermediates, phenylpropanoid metabolism synthesizes a diverse array of secondary metabolites. Many genes and metabolites associated with phenylpropanoid pathways play a key role in feedstock quality and they were identified. Among these, the abundantly marked genes/metabolites in AP13 and VS16 for both H3K9_me2_ and H4K12_ac_ include hydroxycinnamoyl-CoA:shikimate hydroxycinnamoyl transferase (HCT), cinnamyl alcohol dehydrogenase (CAD), phenylalanine ammonia-lyase (PAL), 4-coumarate: CoA ligase (4CL), Cinnamate 4-hydroxylase (C4H), and caffeoyl-CoA 3-O-methyltransferase (CCoAMOT) (Table 4).

### Peaks associated with stress-related genes

Even though we did not impart any stress to derive the reference epigenomes in this study, it was interesting that many biotic and abiotic stress tolerance genes were prominent and inherently marked between AP13 and VS16 genotypes. In total, we identified histone marks on genes related to heat tolerance (176), drought tolerance (9), salinity tolerance (8), cold tolerance (3), flood tolerance (352), heavy metal tolerance (57), and disease resistance (277) in both genotypes (Additional file 1: Tables S6, S7, S8, S9, S10, S11, S12, S13, S14, S15, S16, S17, S18, S19, S20, S21, S22, S23, S24, S25, S26, S27, S28, S29, S30, S31, S32 and S33). For example, four drought-related genes significantly enriched in this study were drought-responsive family protein, DROUGHT SENSITIVE 1, chloroplast drought-induced stress protein of 32 kDa, and drought-induced 19 protein. A representative list of significantly marked (H3K9_me2_ and H4K12_ac_) stress-related genes are highlighted (Table 5).

**Table 5:** List of abiotic and biotic stress-related genes for H3K9_me2_ and H4K12_ac_ modifications in switchgrass genotypes AP13 and VS16

	Number of genes			
	H3K9_me2_		H4K12_ac_	
	AP13	VS16	AP13	VS16
Heat Tolerance Genes				
DNAJ heat shock family protein	115	111	126	199
Heat shock proteins (HSPs)	78	71	91	119
DNAK-related heat shock proteins	22	19	24	21
HEAT repeat-containing protein	10	6	11	7
Heat shock transcription factors	10	22	15	16
Drought Tolerance Genes				
Drought-responsive family protein	5	5	6	7
DROUGHT SENSITIVE 1	3	3	2	1
Chloroplast drought-induced stress protein of 32 kDa	1	1	2	1
Drought-induced 19 protein	5	5	5	7
Salinity Tolerance Genes				
Salt tolerance Zinc finger	3	2	4	2
Low temperature and salt responsive protein family	3	7	7	2
Salt tolerance homolog 2	3	2	6	2
Cold Tolerance Genes				
Cold regulated gene 27	2	1	0	1
Cold, circadian rhythm, and rna binding 2	2	1	2	1
Cold shock domain protein 1	1	1	1	1
Soybean gene regulated by cold 2	0	2	0	1
Cold shock domain protein 3	0	1	0	2
Cold regulated 314 inner membrane 1	0	1	0	2
Cold regulated 413 plasma membrane 1	0	1	0	0
THO complex, subunit 5	0	0	0	1
Flooding Tolerance Genes				
Subtilase family protein	57	28	69	88
Leucine-rich repeat transmembrane protein kinase	28	25	34	44
Transducin family protein/WD-40 repeat family protein	19	22	21	46
Expansin	24	17	21	41
Adenine nucleotide alpha hydrolase-like superfamily protein	25	13	12	123
Heavy metal Tolerance Genes				
Heavy metal transport/detoxification superfamily protein	58	38	52	40
Copper transport protein family protein	6	6	6	6
Heavy metal atpase	3	5	3	3
Chloroplast-targeted copper chaperone protein	3	3	3	6
Farneslated protein 3	1	2	5	4
Farneslated protein 6	1	0	1	0
Disease-Resistance Genes				
NBS-LRR disease resistance protein	68	50	59	96
LRR-NB-ARC domains-containing disease	44	38	38	66
NB-ARC domain containing protein	202	215	251	66
Disease resistance protein (CC_NBS-LRR class)	39	37	35	40
Disease resistance responsive (dirigent-like protein) family protein	21	18	32	27

### Peaks associated with transcription factors

In total, 653 and 650 significantly enriched peaks were annotated as TFs and were methylated and acetylated, respectively, in AP13, while 431 and 579 significantly methylated and acetylated peaks, respectively, were annotated as TFs in VS16. Among these, 17 TFs were differentially marked (H3K9_me2_ and H4K12_ac_) between the two genotypes (Table 6; Additional file 1: Tables S34, S35, S36 and S37). The most prominent TFs in descending order were Myeloblastosis (Myb), Myb-domain containing proteins, Gibberellic Acid Insensitive (GAI)-Repressor of Ga1 (RGA)-Scarecrow (SCR) (GRAS) family, Basic Leucine Zipper Domain (bZIP) family, GATA TF family protein (ability to bind to the DNA sequence GATA), W-box Containing TF (WRKY) family, TEOSINTE BRANCHED1 (TB1) from maize (*Zea mays*), CYCLOIDEA (CYC) from snapdragon (*Antirrhinum majus*), as well as PROLIFERATING CELL NUCLEAR ANTIGEN FACTOR1 (PCF1) and PCF2 (TCP) family, heat shock TFs, Plant AT-rich Sequence and Zinc-binding Protein (PLATZ) TF family protein, and the Basic Helix-Loop-Helix (bHLH) family of TFs.

**Table 6:** Number of transcription factors associated with H3K9_me2_ and H4K12_ac_ modifications in switchgrass genotypes AP13 and VS16

		Number of TFs			
Family of TFs	Function	H3K9_me2_		H4K12_ac_	
		AP13	VS16	AP13	VS16
Myb	Development, differentiation, metabolism, response to abiotic and biotic stresses	103	58	95	82
WRKY	Growth and development, secondary metabolism, leaf senescence, response to abiotic and biotic stresses	37	22	27	38
GRAS	Growth and development, gibberellin synthesis, cell division and endodermic specification	65	38	52	40
bHLH	Plant cell and tissue development, activation of genes in response to light, modulate gene expression, regulation of BR signaling pathway	16	9	20	14
bZIP	Photomorphogenesis, leaf and seed formation, energy homeostasis, response to abiotic and biotic stresses	62	41	68	74
TCP	Development, DNA binding, Morphogenesis, seed germination, leaf senescence, defense response	27	14	19	19
GATA	Nitrogen control, siderophore biosynthesis, photomorphogenesis, circadian regulation, mating-type switching	41	21	61	41
E2F TF3	Cell cycle pathway, transcription activators and repressor, DNA replication	2	0	0	0
E2F FTF	Same as E2F TF3 functions	0	4	6	4
Redox Responsive TFs	Photosynthesis, Activation of gene expression, cross tolerance to abiotic and biotic stresses	2	1	0	0
Heat Shock TFs	Regulation of cellular homeostasis, promote survival and gene expression under environmental factors	21	10	11	15
Global TF Group A2	Regulation of stress-responsive genes and *Hsps*	6	4	5	10
FRS TF	Same as Group A2 TF functions	5	7	10	5
PLATZ TF Family Protein	Response to abiotic and biotic stimuli, seed germination, transcriptional repression, cell cycle regulation and cell division	17	21	11	13
Ethylene Responsive Element Binding Factor 13	Growth and development, hormonal regulation, development of ovule and seed coat	2	5	2	4
TF IIIC Subunit 5	Transcription initiation, RNA Pol III binding	5	4	12	9
Plant-Specific TF YABBY Family Protein	Regulation of defense response, leaf senescence, trichome development, biosynthesis of specialized metabolites	7	7	6	6

### Quantitative PCR validation

**Fig. 4:**
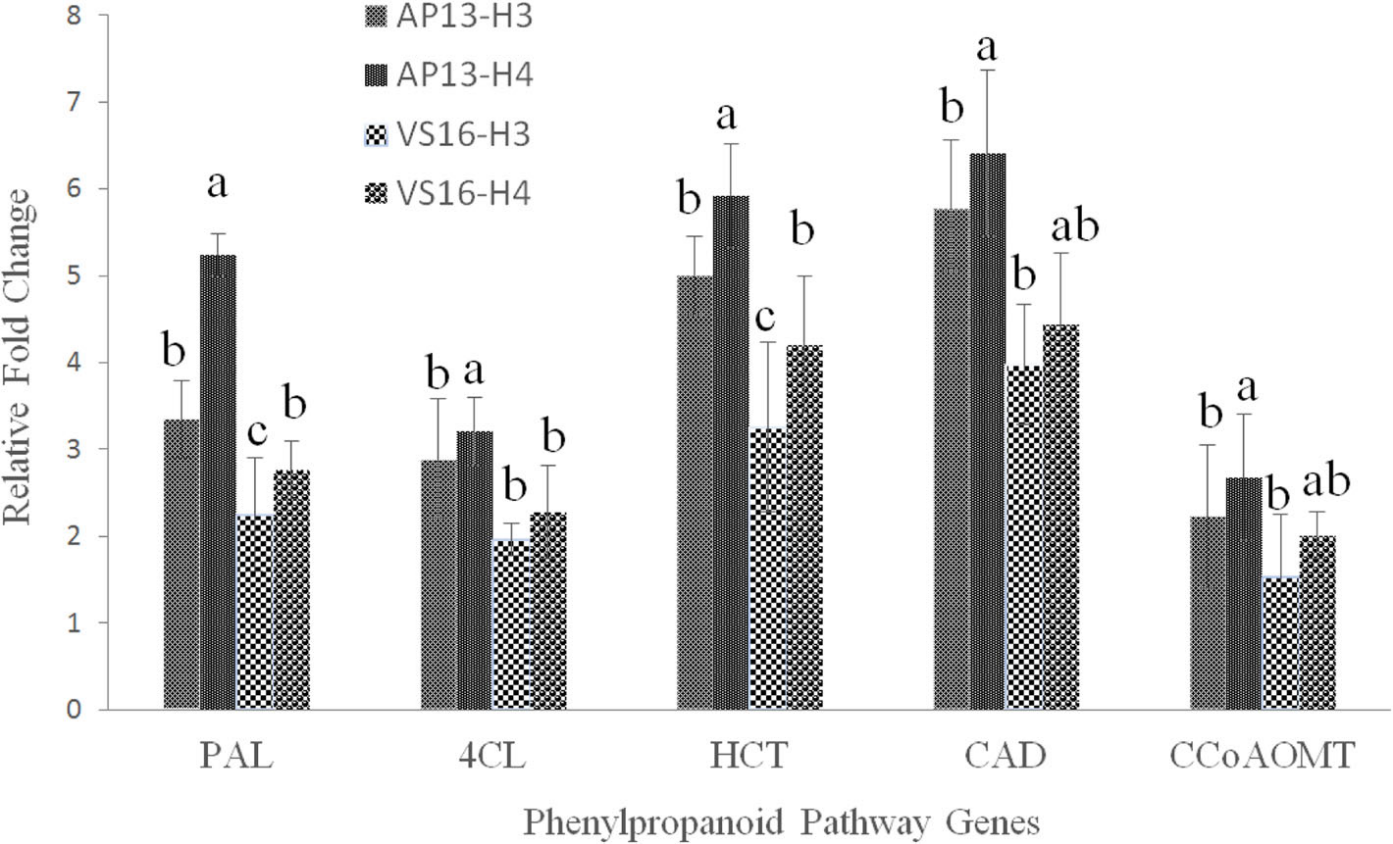
Differential expression of genes between AP13 and VS16 in H3K9_me2_ and H4K12_ac_ modifications using Quantitative-Real-Time PCR (qPCR) analysis. The normalized CT values (ΔΔCT) from qPCR analysis were collected and analyzed by using Minitab 17, and the expression results were presented as mean ± SE. One-way ANOVA was performed on qPCR experiments for multiple comparisons between the mean of samples

Genome-wide ChIP-Seq analysis revealed 17,364 and 13,878 genes associated with peaks between AP13 and VS16, respectively, for both modifications (H3K9_me2_ and H4K12_ac_). Among these, five genes (PAL, 4CL, HCT, CAD, and CCoAOMT) from the phenylpropanoid–monolignol pathway, differentially marked with histone modifications, were selected to confirm the expression level by qPCR. The significant (*P* < 0.05) variations, in the relative expressions, of all five genes indicated that they were more marked in AP13 than in VS16. Further, for both H3K9_me2_ and H4K12_ac_ in these two genotypes the levels of expression were higher for CAD and HCT, while lower for CCoAOMT (Fig. 4).

## Discussion

Histone modifications, such as H3K9_me2_, H4K12_ac_, H3K27_me2_/_me3_, H3K27_me1_, H3K4_me3_, and H3K9_me3_, bind either to heterochromatic or euchromatic regions and orchestrate epigenetic regulation of gene expression in a tissue-specific or genome-wide manner. These modifications have been suggested to play an important role in growth, development, biotic stresses, and abiotic stresses in plants [8, 11–13]. Similar to our previous study in common bean [8] and other studies in rice [7], Arabidopsis [41], cotton [42], and a diatom (*Phaeodactylum tricornutum*) [43], in this study, a genome-wide screening to understand the binding of two histone marks associated with gene inactivation (H3K9_me2_) and activation (H4K12_ac_) was performed using ChIP-Seq analyses in two diverse switchgrass genotypes (AP13 and VS16) belonging to two ecotypes. The percentage of mapped reads to the reference genome in rice ranged between 91.70–98.90% [7], while in switchgrass we obtained mapping rates of 82.32 and 73.42% for H3K9_me2_ as wells as 81.20 and 68.43% for H4K12_ac_ in AP13 and VS16, respectively, which are attributed to the genotype-specific adaptations, variations in GC-content, repeat regions, genome duplications and ploidies [6].

### Histone modification patterns in switchgrass

With only slight variations between the two genotypes, genome-wide histone modification patterns were similar (Additional file 2: Figure S3A-D) when compared to gene expression data from our recent effort [30]. More genes were associated with peaks marked with H4K12_ac_ than H3K9_me2_ in both genotypes, which is attributed to the activation of genotype-specific genes (Additional file 2: Figure S1). Even though the overall distribution pattern of histone marks is similar, slight variations existed between the two genotypes, which is reflected in the distribution of peaks in the respective genomes. Also, more genes were marked with H4K12_ac_ than H3K9_me2_ in both the genotypes. Moreover, bivalent histone modifications were commonly marked on specific genes for both genotypes, suggesting a significant degree of overlap between these two epigenetic marks. Our findings are in agreement with genome-wide histone modification studies in rice [7, 44] and barley [45]. For example, in rice both activating (H3K4_me3_) and repressive (H3K27_me3_) histone modifications formed strong peaks within 1 kb downstream of the TSSs [7, 46]. ChIP-Seq analysis in *Populus* revealed that ~ 90% of ARK1 and ARK2 binding regions were within 1 kb of the TSSs [47]. In rice the enrichment of histone acetylation and methylation marks showed at 500 bp downstream from the TSSs [48]. The H3K4_me3_ distribution showed a clear peak around 300 bp downstream of the TSS in *Arabidopsis* [49]. Additionally, in *Arabidopsis* H3K9_me2_ and H3K4_me_ enrichment spanned around the entire gene body [50, 51].

Previous reports in rice [46] and humans [52] demonstrated that active histone marks are more enriched in bidirectional promoters compared to unidirectional promoters. A detailed analysis of the genomic locations associated with the peaks for H3K9_me2_ and H4K12_ac_ indicated higher affinity levels of H4K12_ac_ to switchgrass genes rather than H3K9_me2_, suggesting the role of active histone marks in transcriptional activation of switchgrass genes. However, a greater number (> 10%) of peaks associated with H4K12_ac_ and H3K9_me2_ marks spanned 5 kb upstream and downstream from TSSs in AP13 when compared to VS16.

Recently, the preferential binding of TFs (~ 86%) between − 1000 to + 200 bp from a TSS has been identified in *A. thaliana* [53]. In *Populus*, the majority of binding regions for all TFs analyzed have shown a general preference for binding near genes, and they were specifically enriched within 5 kb of the TSSs [47]. Histone-mediated transcription initiation/inactivation occurs by binding to promoter and enhancer regions and also by modulating transcription elongation [48]. Analysis of histone modification profiles with bins of increasing gene length in *Arabidopsis* revealed that most peaks have been localized around 480 bp downstream of the TSSs. The shape and width of the H3K36_ac_ peaks were independent of gene length [12]. Similarly, in this study most H3K9_me2_ and H4K12_ac_ peaks were localized between 0 and 499 bp downstream, supporting the idea that peak position, shape, and length are independent of gene length. Transcription factors and DNA binding proteins were highly enriched in the cluster of genes associated with H3K27_me3_ in tissue-specific repression as reported in *Arabidopsis* (6). Genome-wide comparative analyses using two histone marks, H3K9_ac_ and H3K4_me3_, in *A. thaliana* and *A. arenosa* revealed coordination between histone modifications and gene expression within and between these species, including allopolyploids [6].

### Pathways and genes related to biomass/biofuel production

**Fig. 5:**
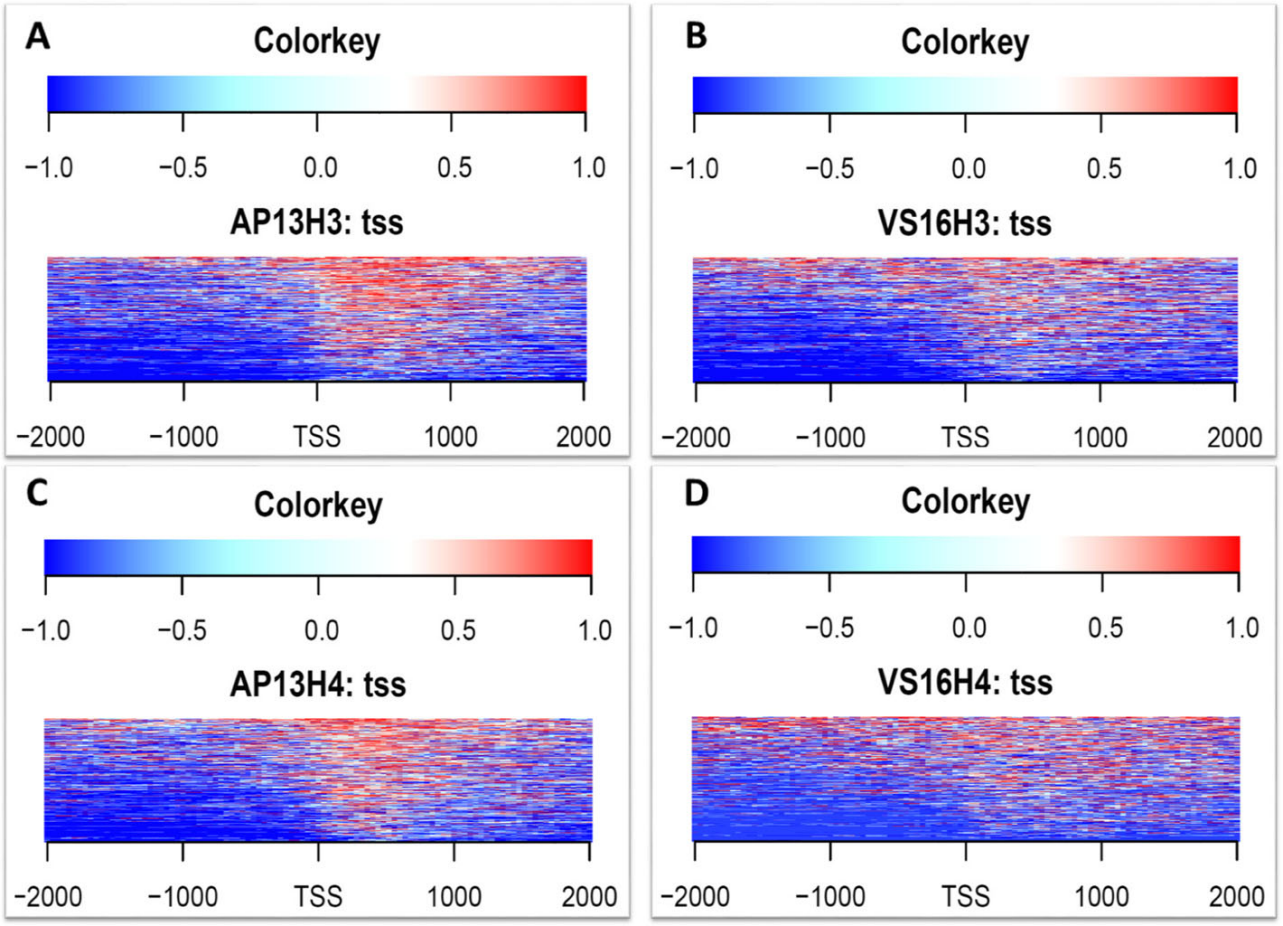
Differentially marked histone modifications (H3K9_me2_ and H4K12_ac_) on 430 selected phenylpropanoid-monolignol pathway-related genes between AP13 and VS16 using heatmap analysis

Further, we narrowed our search to identify histone markings on genes related to C4-photosynthesis andphotorespiration pathways, and phenylpropanoid-monolignol pathway, as they play a key role in biomass and biofuel production [54]. Using three histone acetylation marks (H3K9_ac_, H4K5_ac_, and H3K18_ac_) and two histone methylation marks (H3K4_me3_ and H3K4_me2_) and targeting five C4 genes (CA, PPDK, ME, PEPCK, and RBCS2), comparative ChIP-Seq analyses reported in maize, sorghum (*Sorghum bicolor*), and foxtail millet (*Setaria italica*) revealed a role for histone modifications in light-specific and cell type-specific responses [55]. Supporting this idea, our analyses here showed preferential binding of histone marks (H3K9_me2_ and H4K12_ac_) between AP13 and VS16 in five prominent C4-pathway genes (DIT, PEP, PEPC, BASS and CA) five photorespiration-related genes (2-OG, Glu, 3-PGA, RuBisCo, and GLN); and, six monolignol pathway-related genes (C4H, PAL, 4CL, HCT, CAD, and CCoAOMT). Genes that mediate phenylpropanoid pathways play a key role in feedstock quality. A recent histone binding study in *Eucalyptus grandis* reported that about 8% of phenylpropanoid pathway genes were marked by H3K4_me3_ [56]. In this study, the histone modification patterns for 430 monolignol pathway-related genes between two ecotypes were mostly correlated (Fig. 5), supporting the idea of a common histone modification code between genotypes and possibly across species, as has been suggested.

Interestingly, this study identified several abiotic and biotic stress-related genes differentially marked by H3K9_me2_ and H4K12_ac_ in VS16 and AP13, which may contribute to differing genetic potential of these two contrasting ecotypes (upland and lowland). Here, we identified H3K9_me2_ and H4K12_ac_ modifications on different stress-related genes abundantly marked in both genotypes such as disease-resistance, heat tolerance, and flood tolerance genes. Our recent transcriptome analyses in switchgrass genotypes AP13 and VS16 identified over 200 significantly enriched stress-related genes [8]. Similarly in this study, prominently associated with H3K9_me2_ and H4K12_ac_ were genes that encode the leucine rich repeats (LRR)-family of proteins, NBS-LRR, NB-ARC, LRR-NB-ARC, and CC-NBS-LRR (disease resistance); the heat shock proteins (HSPs), DNAJ and DNAK (heat tolerance); and the subtilase family protein, LRR-transmembrane protein kinase and adenine nucleotide alpha hydrolase-like superfamily protein (flooding tolerance) (Table 5). Even though the overall binding patterns were similar, slight variations in markings were observed between the two ecotypes. Therefore, changes noticed in histone bindings represent inherent differences between the upland (VS16) and lowland (AP13) switchgrass ecotypes.

### Quantitative PCR validation

It is important to validate differentially marked genes in switchgrass, a prominent biofuel crop, especially in regard to the phenylpropanoid–monolignol biosynthesis pathway. Previous reports suggested that over ten different genes play an important role in the phenylpropanoid–monolignol pathway in switchgrass [57–59], including the five genes selected in this study (PAL, 4CL, HCT, CAD, and CCoAOMT). Though transcriptional networks associated with the phenylpropanoid pathway have been reported [60], studies regarding the regulation of phenylpropanoid genes by histones in plants are limited. In maize, acetylation of H3K9/K14 in the promoter region resulted in activation of the anthocyanin biosynthetic gene, A1, by interacting with EMSY-related factor, thus linking TFs with chromatin modifications [61]. Our results suggested that all five genes selected for ChIP-qPCR from the phenylpropanoid pathway were significantly marked by both histone modifications in both genotypes. Recently, comparative RNA-Seq analyses in switchgrass suggested that phenylpropanoid pathway genes were abundantly expressed in crowns and rhizomes of lowland cultivar, Kanlow, when compared to upland cultivar, Summer [62]. Similarly, comparative ChIP-Seq and ChIP-qPCR analyses as reported here in switchgrass suggest that differentially methylated and acetylated histone modification regions associated with the phenylpropanoid pathway genes were relatively abundant in lowland cultivar, AP13, when compared to upland cultivar, VS16. This suggests the role of active and inactive histone modification marks in regulating genes associated with biomass yield and biofuel production. Further, it is reasonable to assume that phenylpropanoid pathway genes may undergo a combinatorial transcriptional regulation involving TFs and histone modifications.

## Conclusions

Our genome-wide screening for histone methylation (H3K9_me2_) and acetylation (H4K12_ac_) marks in two contrasting ecotypes of switchgrass identified similarities and disparities between AP13 and VS16 at the epigenome level. The distribution of significantly enriched peaks spanned 0–2000 bp around TSSs, and most of these peaks were observed in non-genic regions. Our comprehensive analysis of annotated peaks facilitated the identification of key regulatory marks, genes, and TFs involved in biosynthetic pathways, including C4 photosynthesis, photorespiration, phenylpropanoid–monolignol, as well as biotic and abiotic stresses.

In summary, we integrated ChIP-seq and RNA-seq datasets to explore the interactions between histone modifications and TFs/transcripts related with gene expression and their patterns across two contrasting genotypes of switchgrass, AP13 and VS16. Furthermore, the integrated methodology and results highlighted here will guide future studies in exploring similar interactions in gene expression and regulation.

## Additional files

Additional file 1: **Tables S1-S37.** Supplementary tables with legends in the first sheet called “Supplementary Table Legends”. (XLSX 479 kb)

Additional file 2: **Figure S1.** Number of H3K9_me2_ and H4K12_ac_ binding regions in switchgrass genotypes AP13 and VS16. (PPTX 320 kb)

Additional file 3: **Figure S2.** Heatmap of preferentially marked H3K9_me2_ and H4K12_ac_ genes along with transcriptional activity 2 kb upstream and downstream from TSSs in switchgrass genotypes AP13 and VS16. (**A)** H3K9_me2_-AP13 (**B)** H3K9_me2_-VS16 (**C)** H4K12_ac_-AP13 and (D) H4K12_ac_-VS16. Genes were sorted according to their expression level from mRNA analysis. The histone modification intensities are displayed along with − 2 kb to + 2 kb regions around TSSs. (PPTX 1474 kb)

Additional file 4: **Figure S3.** Peak width distributions of H3K9_me2_ and H4K12_ac_ modifications in switchgrass genotypes AP13 and VS16. (**A)** H3K9_me2_-AP13 (**B)** H3K9_me2_-VS16 (**C)** H4K12_ac_-AP13 and (D) H4K12_ac_-VS16. The numbers 499 and 999 in peak width bin column refer to the number of peaks having a peak width between 0 to 499 and 500 to 999, respectively. (PPTX 369 kb)

Additional file 5: **Figure S4.** List of epigenomic factors with H3K9_me2_ and H4K12_ac_ modifications in switchgrass genotypes AP13 and VS16. (PPTX 325 kb)

## Data Availability

All data sets generated as part of this study are available at the SRA section of the NCBI with the bio-project number PRJNA280864 for ChIP-Seq experiment.

## Abbreviations

2-OG: 2-oxoglutarate
3-PGA: 3-phosphoglycerate
4CL: 4-coumarate: CoA ligase
BASS: Bile acid sodium symporter/pyruvate transporter (BASS)
bHLH: The Basic Helix-Loop-Helix
BS-Seq: Bisulfite sequencing
bZIP: Basic Leucine Zipper Domain Containing TF
C4H: Cinnamate 4-hydroxylase
CA: Carbonic anhydrase (CA)
CAD: Cinnamyl alcohol dehydrogenase
CAT: Catalase
CCoAMOT: Caffeoyl-CoA 3-O-methyltransferase
ChIP-Seq: Chromatin immunoprecipitation sequencing
CT: Cycle threshold
DIT: Dicarboxylate transporter
FDR: False discovery rate
GLN: Glutamine
GLU: Glutamate
GO: Gene Ontology
GRAS: Gibberellic Acid Insensitive (GAI)-Repressor of Ga1 (RGA)-Scarecrow (SCR)
GS: Glutamate synthase
H3K9_me2_: Histone H3 di-methylated at lysine 9
H4K12_ac_: Histone H4 acetylated at lysine 12
HCT: Hydroxycinnamoyl-CoA:shikimate hydroxycinnamoyl transferase
IGV: Integrative Genomics Viewer
KEGG: Kyoto Encyclopedia of Genes and Genomes
lncRNAs: Long non-coding RNAs
Log_2_FC: Fold change
MeDIP-Seq: Methylated DNA immunoprecipitation sequencing
Myb: Myeloblastosis
NADP: Nicotinamide adenine dinucleotide phosphate
PAL: Phenylalanine ammonia-lyase
PEP: Phosphoenol pyruvate
PEPC: Phosphoenolpyruvate carboxylase
PEP-CK: Phosphoenolpyruvate carboxykinase
PLATZ: Plant AT-rich Sequence and Zinc-binding Protein
qPCR: Quantitative Real-Time (RT)-PCR
QTL: Quantitative trait loci
RuBisCo: Ribulose 1,5-bisphosphate carboxylase/oxygenase
SICER: Spatial clustering for identification of ChIP-enriched regions
siRNAs: Small interfering RNAs
TCP: TEOSINTE BRANCHED1 (TB1), CYCLOIDEA (CYC), PROLIFERATING CELL NUCLEAR ANTIGEN FACTOR1 (PCF1) and PCF2
TEs: Transposable elements
TF: Transcription factor
TSS: Transcriptional start site
WRKY: W-box Containing TF
