# Supplementary figures and images for: Genome-wide identification of histone methylation (H3K9me2) and acetylation (H4K12ac) marks in two ecotypes of switchgrass (Panicum virgatum L.)
Source: BMC Genomics. 2019 Aug 22;20:667. doi: 10.1186/s12864-019-6038-x (PMC6704705; doi:10.1186/s12864-019-6038-x)

## Slide 1
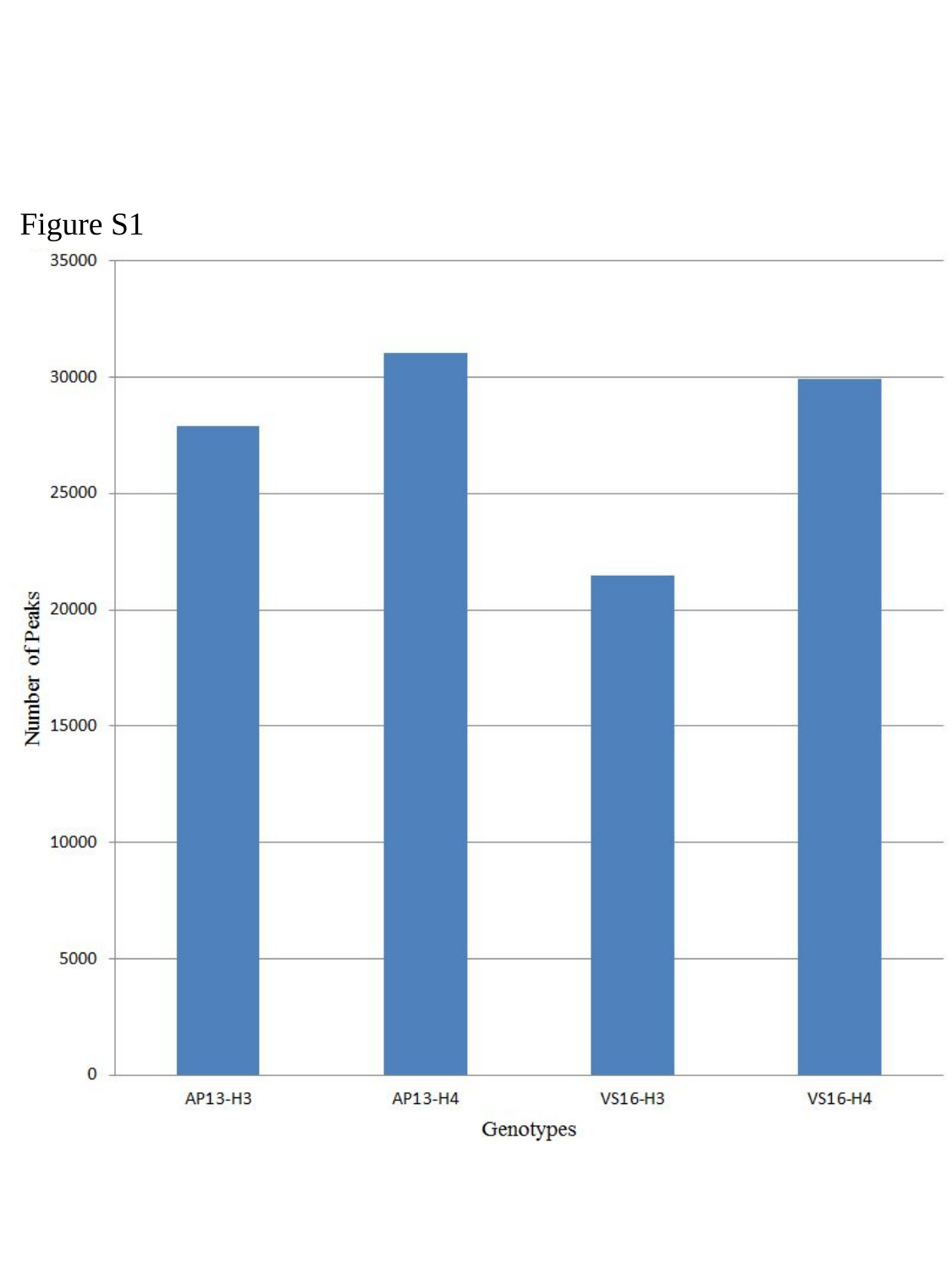

Figure S1

Supplement: Supplementary file 2 — Figure S1. Number of H3K9me2 and H4K12ac binding regions in switchgrass genotypes AP13 and VS16. (PPTX 320 kb) [file 12864_2019_6038_MOESM2_ESM.pptx]

## Slide 1
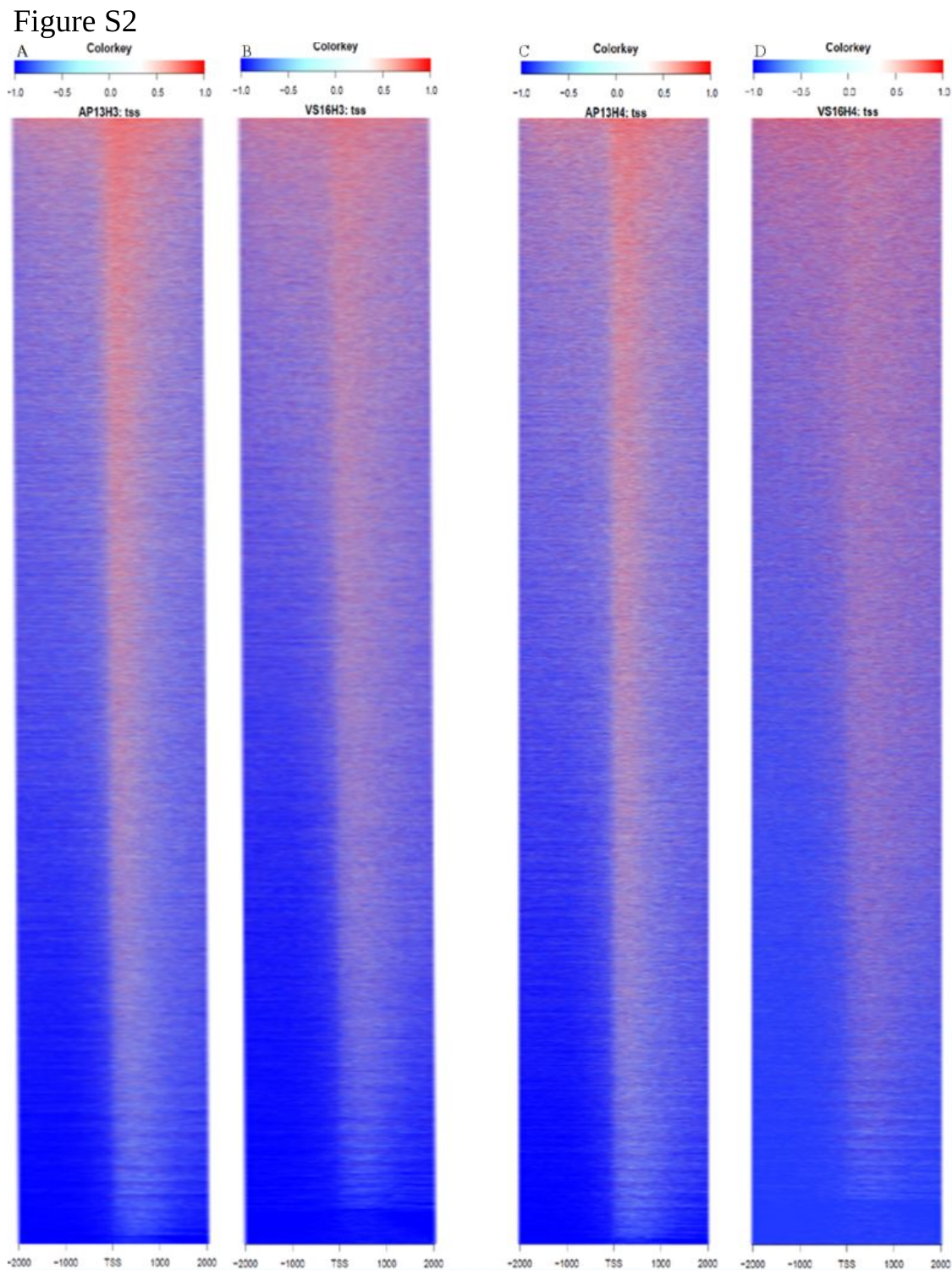

Figure S2

Supplement: Supplementary file 3 — Figure S2. Heatmap of preferentially marked H3K9me2 and H4K12ac genes along with transcriptional activity 2 kb upstream and downstream from TSSs in switchgrass genotypes AP13 and VS16. (A) H3K9me2-AP13 (B) H3K9me2-VS16 (C) H4K12ac-AP13 and (D) H4K12ac-VS16. Genes were sorted according to their expression level from mRNA analysis. The histone modification intensities are displayed along with − 2 kb to + 2 kb regions around TSSs. (PPTX 1474 kb) [file 12864_2019_6038_MOESM3_ESM.pptx]

## Slide 1
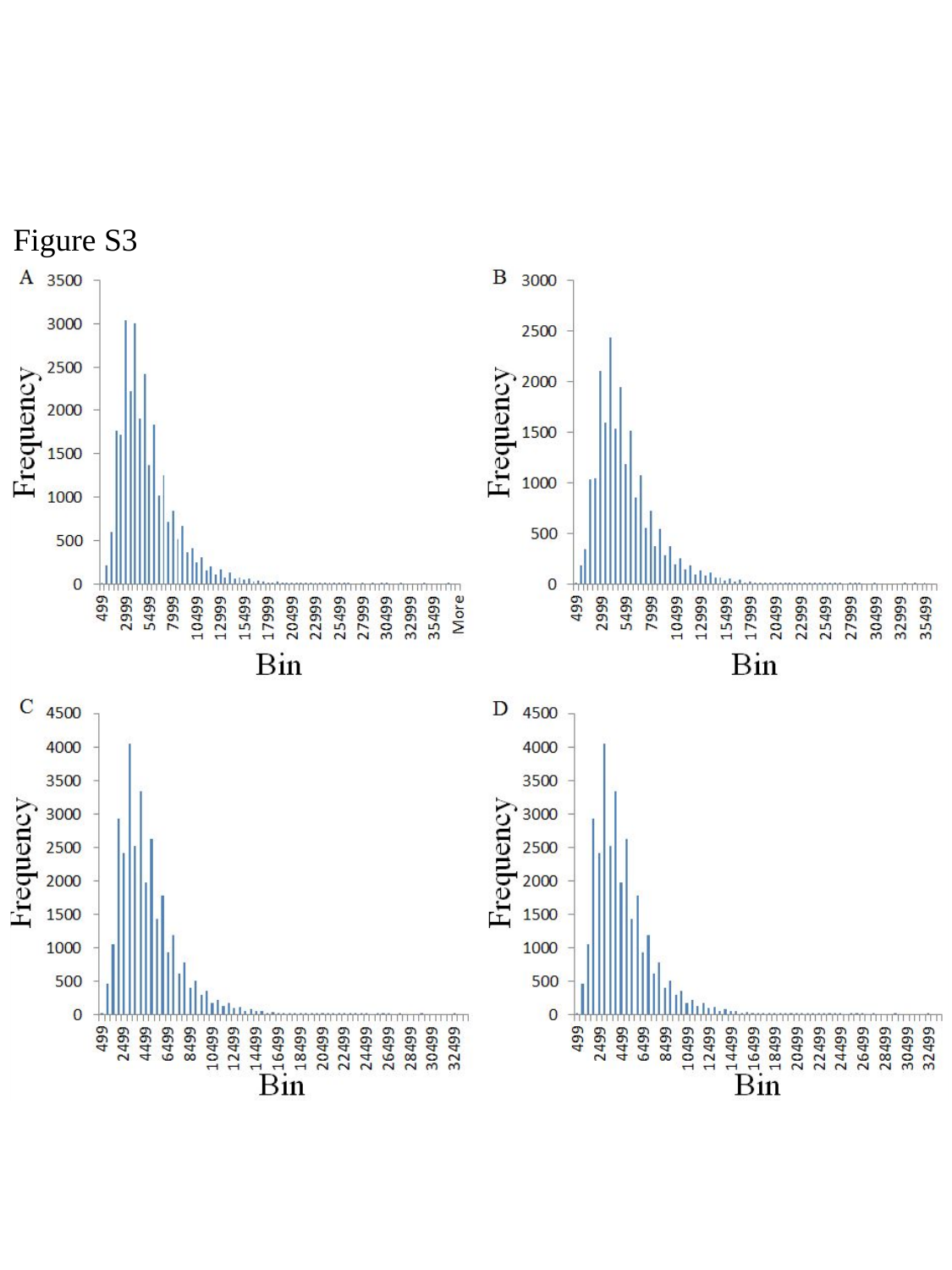

Figure S3

Supplement: Supplementary file 4 — Figure S3. Peak width distributions of H3K9me2 and H4K12ac modifications in switchgrass genotypes AP13 and VS16. (A) H3K9me2-AP13 (B) H3K9me2-VS16 (C) H4K12ac-AP13 and (D) H4K12ac-VS16. The numbers 499 and 999 in peak width bin column refer to the number of peaks having a peak width between 0 to 499 and 500 to 999, respectively. (PPTX 369 kb) [file 12864_2019_6038_MOESM4_ESM.pptx]

## Slide 1
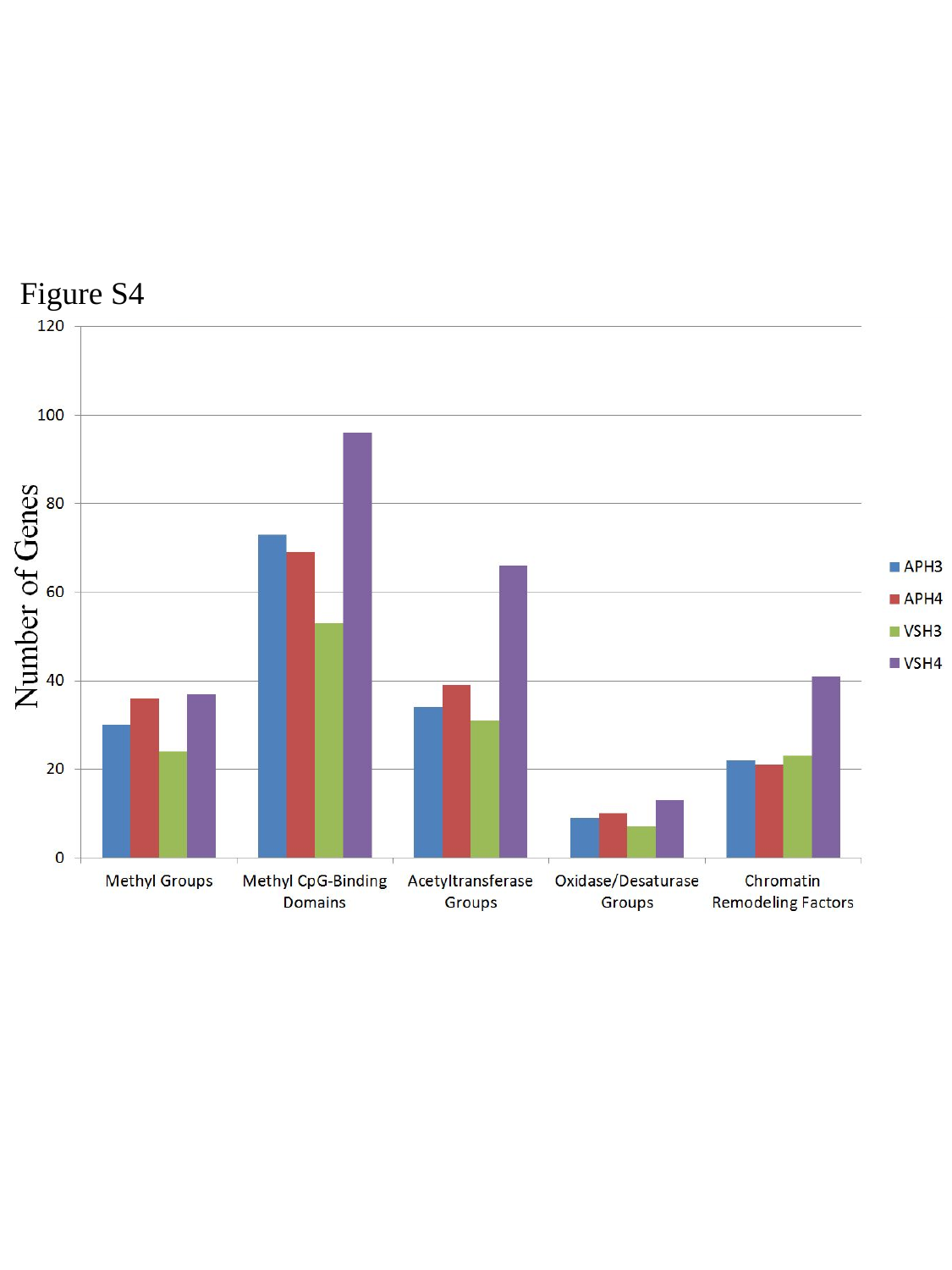

Figure S4

Supplement: Supplementary file 5 — Figure S4. List of epigenomic factors with H3K9me2 and H4K12ac modifications in switchgrass genotypes AP13 and VS16. (PPTX 325 kb) [file 12864_2019_6038_MOESM5_ESM.pptx]
